# Editorial

**Published:** 1861-02

**Authors:** 


### EDITORIAL.

*Report of the Committee on Medical Education, presented to the Academy of Medical Sciences, Jan. 4th,* 1861. By N. S. Davis, M. D., *Chairman of the Committee.*

According to the by-laws of the Academy, the Committee on Medical Education are requested to "take cognizance of the systems of Medical Education, private and public, in this city and State, as compared with such as are elsewhere in use," &c., &c.; and are requested to report "at least once a year to the Academy."

In fulfilling the duties thus imposed, the Chairman of your Committee would respectfully submit the following report:

In regard to Medical Colleges, we would report that there are two in the State, both of which are located in this city. Several years since attempts were made to establish Medical Colleges at Jacksonville, at St. Charles, and at Rock Island, but without success.

The Rush Medical College of this city, with an independent charter from the State Legislature, commenced an active existence in 1842, and has continued in successful operation to the present time. Its plan of instruction is identical with that adopted by a majority of the Medical Colleges in this country; consisting of one annual College term of sixteen weeks, during which all the branches of medical science and art are taught by lectures and demonstrations. Its requisitions for graduation are the same as those usually adopted, viz: three years study, two courses of lectures, during at least one of which, the candidate must have attended to Practical Anatomy and Hospital Clinical instruction; the attainment of twenty-one years of age; the possession of a good moral character; and the ability to sustain an examination in the several branches taught.

During the past history of the Rush Medical College we believe, it has adhered practically to these regulations as faithfully as any Medical College in our country. Its several Chairs have been occupied by men of talent, some of whom, like the professors of Surgery and Chemistry, have held their position from the foundation of the school to the present time, and have attained a high reputation in their respective departments.

The prosperity of the school has been maintained with much uniformity. During the last ten years the number of students in attendance has varied from 100 to 150; the latter number being present, if we remember right, during the session of 1856-7. We are informed that the class in attendance during the present session, numbers about 145.

Besides the several courses of lectures embraced in the College curriculum, the students enjoy the advantages of one or two College Clinics each week, and so many of them as choose to take the tickets, have access to the several Hospitals in the city.

The second Medical College in this city, is the Medical Department of Lind University. It was organized in 1859, and its first annual course of instruction was given in the fall and winter of 1859-60; and its second course is now in progress. The plan of organization adopted by this department of the University, differs from that adopted by the great majority of Medical Colleges in this country, in three important particulars, viz: in the length of the annual session; in the greater number of professorships; and in the division of the students into Junior and Senior classes.

The regular annual lecture term is five months; commencing with the first week in October, and ending the first week in March. There are thirteen professorships, viz: one of Descriptive Anatomy; of Inorganic Chemistry; of Physiology and Histology; of Materia Medica; of General Pathology and Public Hygiene; of Surgical Anatomy and operations of Surgery; of Organic Chemistry and Toxicology; of Obstetrics and Diseases of Women; of Practical Medicine; of Practical Surgery; of Medical Jurisprudence; of Clinical Medicine; and of Clinical Surgery. The first five departments named, together with Practical Anatomy in the Dissecting room, constitute the Junior course; while the remaining departments make up the Senior course. In the Junior department there are four lectures daily throughout the term. In the Senior department, there are also four lectures in the College, and one Clinic in the Mercy Hospital, daily, throughout the term. In addition to the daily Hospital classes, there is a Surgical Clinic in the College every Wednesday, and a Clinic on Diseases of Women and Children, every Saturday.

The foregoing plan was adopted by this institution, for the purpose of rendering the study of Medicine more systematic or methodical, by compelling each student to concentrate his attention upon the more elementary branches, until he could sustain a fair examination therein, before he could advance to the more practical departments; and at the same time, by increasing the length of the lecture term and doubling the number of professors, to make the entire field of College instruction much more extensive than in the ordinary College courses. Another prominent object was to elevate the departments of Clinical Medicine and Surgery to their proper position by making them a part of the regular daily course of instruction in the Senior department. The requisites for graduation are the same as we have already stated in reference to Rush Medical College. The number of students who attended the first course in the University was thirty-three; and the present class numbers fifty.

Though this department of the Lind University is not yet two years old, it has made rapid progress in the accumulation of the means of illustration and study. Its Museum already contains 2970 specimens, belonging to the departments of Anatomy, Surgery, Obstetrics, and Pathology. The chemical Labratory is well filled; and the Library contains near 700 volumes. We have thus briefly noticed the two Medical Colleges in our city. We believe they are an honor to the city and the State, and are worthy of the fostering care of the profession and of the community generally.

*Private Instruction.*—Previous to the organization of the Medical Department of the Lind University in 1859, no arrangements had been made in this city for the systematic instruction of Medical Students, during the interval from the close of one lecture term to the opening of the next. But the feelings of rivalry engendered by the organization of a new school, soon caused an organization to be effected under the auspices of the Rush Medical College, consisting of six or eight of the more enterprising and active practitioners of this city, for the purpose of systematic summer instruction; embracing examinations, lectures, and clinics. Its plans were carried into successful practical operation during the summer of 1860, at least, from the first of March to the first of July.

The faculty of the Medical Department of Lind University also adopted, as a part of their system of medical instruction, arrangements for a systematic course of summer study. This plan was so arranged that the students would have one examination, with familiar explanations, and one clinical lecture each day, throughout the whole interval, from one lecture term to the next. This plan was carried out by the faculty, and attended by a regular class of students during the past summer.

*Hospitals.*—There are, at present, three hospitals in the city, in each of which clinical instruction is given to some extent; and each of which, consequently, becomes an important aid to the education of the profession.

And although these institutions are small, compared with some of those in the older eastern cities, yet we doubt whether there is a city in the Union, in the Hospitals of which, a greater amount of clinical and practical instruction is given at the bedside of the sick, than those of our own city.

If the foregoing representations are correct, it will be seen that we have in our midst, not only two well organized Medical Colleges, but also all the facilities both for private and clinical instruction, that can be found elsewhere; and consequently, that Chicago is not more a centre of commerce, than a seat of medical instruction. It was feared by many in the profession, that the organization of a second College here, would merely divide the patronage that had hitherto hardly been sufficient to sustain the first, and thereby prove an injury to the profession. Experience has shown those fears to have been groundless. On the contrary, the organization of the new school, has incited the faculty of the old one to greater exertions, to the organization of private courses of instruction, to more extended hospital and clinical advantages, and consequently they have drawn to their lecture rooms an increased number of students. Hence, instead of one school maintaining instruction sixteen weeks annually, to a class ranging from 110 to 150, as was the case prior to 1859, we have now two schools, with collateral organizations for medical instruction all the year, the one with a class of 145, and the other of 50, making an aggregate of 195 students now receiving instruction in the city. And we predict, that if the faculty of each school, shall continue a liberal and honorable emulation; each striving honestly to make the institution to which they are attached, worthy of the patronage of the profession, five years will not elapse before the aggregate number of students assembling annually for medical instruction in our city, will reach from three to four hundred,

One of the greatest obstacles in the way of elevating the standard of medical education, is, the inordinate hurry of students to enter upon the practical duties of the profession. Many of them can hardly be induced to read a work on Anatomy before they become impatient to take up a work on practice. So they can scarcely get through one College course of lectures, before they must enter upon practice. We hold it to be the duty of every member of the profession, as well as of the Colleges, to discourage this tendency, and to insist on a full period of study, and a full share of that study to be devoted to the fundamental branches of medical science. All of which is respectfully submitted.

---

Anesthetics.—The following letter from Dr. Prince will be read with interest:

Jacksonville, Jan. 17th, 1861.

*Prof. Davis*—Dear Sir: In reply to your "request" in the December number of the Examiner, I reply that I commenced the use of Chloroform in Surgical operations and in cases of parturition immediatly upon its introduction by Prof. Simpson. I had been previously using Ether, and I was at once greatly pleased with the superiority of Chloroform in its pleasantness to patients, and the speediness and completeness of its effects. I never saw any unhappy results from its use, but I soon became alarmed by the multiplied cases of fatal results, reported in newspapers and medical journals.

In 1850 I found Dr. Mussey (in Cincinnati,) using a combination of three-fourths of Ether and one-fourth of Chloroform, *by bulk*, and I have used this combination ever since. I have not used unmixed Chloroform for Surgical and Obstetrical cases for ten years. I have sometimes used Ether alone, but not often.

In two cases, respiration ceased while patients were under the influence of this mixture. In the first of these cases, the patient lying upon his side, horizontally, was undergoing an operation for excision of a cancer of the lower lip, and the formation of a new lip. The respiration suddenly ceased. No time was lost in turning the patient over, *to* and *fro*, according to Marshal Hall's "Ready Method," The patient breathed again, and the operation was proceeded with. I, at the time, attributed this to blood in the glottis.

In the next case, the patient was lying upon a bed about to undergo amputation of the leg. The assistant, who was administering the anesthetic, said that the patient did not breathe. The patient was immediately turned upon his side, upon his face, and back upon his side, and then upon his back, repeating this process to and fro about sixteen times a minute, and pausing a little each time the patient came upon his back, side and face. Not many of these semi-revolutions were required, to restore respiration, which commenced with a gap. A methodist minister who was standing by as a spectator, remarked that the patient had been nearly enough "across the river, to hear the horn blow on the other side."

As soon as respiration was completely restored, the operation was proceeded with, more of the anesthetic being administered as it was needed.

*A Fatal Case.*—A dog was being rendered insensible for a Surgical experiment, when his breathing was noticed to cease.

He was immediately turned over to and fro, and the breathing commencing with a gap, went on as before. The experiment was then performed, and the dog made a good recovery. At another time the same dog was undergoing a different experiment, when his breathing was noticed to cease. He would have been instantly turned over as on the former occasion, but that it was a little inconvenient to stop the process for that purpose. The blood did not flow as freely as it was expected to do, and the process, (testing Simpsons *accu-pressure* for controlling hemorrhage,) was suspended to turn the animal over, but to no purpose; too much time had been lost. If the "ready method" had been resorted to immediately on the cessation of respiration, I have no doubt it would have been as effectual as in the other instance with the same dog. A little impatience at being interrupted cost the animal his life.

Without stopping to reason much upon the matter, these propositions must be plain enough:

To secure a *speedy* entrance of air into the lungs is the one thing needful.

To turn the patient over, to and fro is the *speediest* method of securing the one thing needful.

Therefore, this expedient should in all cases be first resorted to.

It should be resorted to in all cases *immediately* upon noticing the cessation of inspiration, at whatever inconvenience.

If the patient is in the sitting posture, he should be *immediately* thrown upon the floor and rolled over.

It follows that a man of caution, who entertains these views, will never give a patient an anesthetic with such surroundings as to render it impracticable to turn him over, to and fro, upon the occurrence of any, (even suspicious) flagging of respiration.

What has been said, leads to the inference that all other restoratives are practically excluded, except those which may be resorted to at the same time.

The patient may be slapped upon the back, and cold water may be dashed upon the face, at the same time that the semi or quarter rotations are being practiced, and they are therefore appropriate enough, as *auxiliaries*, to the "ready method."

What about applying a bottle of ammonia to the nose—of giving the patient a drink of brandy when he is not breathing? How much vapor of ammonia will go into the nostrils in the absence of inspiration, and where will the brandy go more readily than into the larynx under these circumstances?

The *fatal* objection to these or any other remedies which take the place of artificial respiration, is, that these uselessly squander the only few moments, during which respiration can be restored.

Upon the theory that the heart first ceases its function, and the lungs afterwards theirs, the question is, whether the *first few moments* shall be spent in rolling the patient over, thus applying that heart-stimulant, which arises from the continuance of the flow of blood through the lungs, thus draining the heart of blood on one side, and replenishing it with blood upon the other; or whether they shall be spent in getting ready a galvanic apparatus with which to *shock* the patient,—any man sees the decision of the question in its very statement.

The *first few moments* must be employed in keeping up the respiration, and there is but one means of doing this on the instant. The seconds of time diminish in value in a rapid ratio, and the very first second may be employed in turning the patient over—thus securing a change in the amount of air in the lungs, by the necessary motion of the ribs and diaphram during the change of position, and this change in amount of air is respiration.

Upon the theory that the danger arises from paralysis of the muscles of the epiglottis, or upon a loss of sensibility which fails to secure their action, through reflex nervous action, or from obstruction of the fissure of the glottis, by blood, or the viscid secretions, the process of turning over meets the indications coming from all these theories,—even that of the falling of the paralyzed epiglottis upon the glottis; for while the patient is upon his side the ribs will fall together, and the air will rush out, and when he comes upon his face, the epiglottis will fall forward or downward, by its own weight, at the same time that the ribs will expand, and air will rush in.

Sticking a hook into the base of the tongue, and pulling it forward, meets the indication upon the theory of paralysed epiglottis, but unfortunately it must be useless, if respiration has ceased from any other cause.

The process of turning the patient over, meets all the causes existing singly or combined.

I pray the Lord that it may in every case be resorted to *at once.*

Yours for Science,

DAVID PRINCE.

---

*Arseniate of Gold in the Treatment of Cancer.*—M. Massard, strongly recommends the Arseniate of Gold as an internal remedy for the cure of Cancer. We quote the following from the American Medical Monthly:

"Arseniate of Gold is prepared by shaking together a solution of pure Chlorate of Gold, and a solution of Arseniate of Potash, allowing the mixture to stand 24 hours, filtering and drying the precipitate. On account of its insolubility in water, arseniate of gold may be given in the solid form. The maximum dose is two centigrammes a day, in two divided doses. The dose ordinarily commenced with, is three or four milligrammes a day, increasing one milligramme every third day, until the maximum dose is reached, which is to be continued several months. Toxical effects have never yet been observed."

---

*Aromatic Sulphuric Acid in Treatment of Tapeworm.*—Dr. B. Darrach, of Quincy, Ill., publishes in the American Journal of Medical Sciences, some cases showing the efficacy of Aromatic Sulphuric Acid as a remedy for Tapeworm. To be effectual, one drachm of the acid should be given well diluted in water, in the course of three or four hours. On the third or fourth day it may be repeated.

*Chloroform in Congestive Chills.*—The inhalation of chloroform in the cold stage of congestive chills, is confidently recommended by Dr. H. L. Byrd, of Savannah, Ga., in the Oglethorpe Medical and Surgical Journal.

---

*Digitalis in Delirium Tremens.*—Mr. Jones, of Jersey, England, strongly recommends large doses of Tinct. of Digitalis as a remedy for Delirium Tremens. He gave it in ℥ ss doses.

---

### AMERICAN MEDICAL ASSOCIATION.

The fourteenth annual meeting of the American Medical Associatiou will be held, in Metropolitan Hall, city of Chicago; commencing on the *first* Tuesday in June next.

Each regularly organized Medical Society is entitled to send one delegate for every ten of its members; and each Medical College is entitled to two delegates. It is desired that the names of delegates should be forwarded to the undersigned, as soon after their appointment, as practicable.

Chicago, Feb. 1st, 1861. H. A. JOHNSON,
*Assist. Secretary.*

Editors of Medical Journals please copy.

---

### SUMMER COURSE OF MEDICAL INSTRUCTION.

A full course of medical instruction, including Clinics in the Hospital and at the Dispensary, together with Practical Anatomy in the dissecting room, will be given throughout the coming summer, in connection with the Medical Department of Lind University.

The plan will be so arranged as to enable the students to attend one Clinic, and one familiar lecture with examinations, daily, from the end of the present college term on the 1st Monday in March, until the commencement of the next regular term in October.

